# Survival after Cardiac Arrest Secondary to Massive Pulmonary Embolism

**DOI:** 10.1155/2018/8076808

**Published:** 2018-01-31

**Authors:** Abdullah E. Laher, Muhammed Moolla, Feroza Motara, Fathima Paruk, Guy Richards

**Affiliations:** ^1^Department of Emergency Medicine, Faculty of Health Sciences, University of the Witwatersrand, 7 Jubilee Road, Parktown, Johannesburg 2193, South Africa; ^2^Department of Critical Care, Faculty of Health Sciences, University of the Witwatersrand, 7 Jubilee Road, Parktown, Johannesburg 2193, South Africa

## Abstract

**Introduction:**

It is estimated that the diagnosis of pulmonary embolism (PE) is missed in as many as 84% of all cases of PE. Cardiac arrest following PE is generally associated with poor outcomes.

**Case Report:**

A 43-year-old man presented to the Emergency Department (ED) in cardiac arrest. Swelling of his right lower limb was noted on arrival. Point of care ultrasound was performed during ongoing cardiopulmonary resuscitation (CPR) and showed a thrombus in the right iliofemoral vein as well as dilatation of the right ventricle. Fibrinolytic therapy was initiated immediately and a return of spontaneous circulation (ROSC) was achieved 30 minutes later. The diagnosis of PE was finally confirmed on computed tomography pulmonary angiography once haemodynamic stability was achieved. The patient was thereafter transferred to the intensive care unit for postresuscitation care and further management. Several days later, he was discharged home neurologically intact and fully recovered.

**Discussion:**

Since outcomes after cardiac arrest following PE are generally dismal, available and potentially life-saving interventions to restore pulmonary circulation should be rapidly implemented when PE is the likely cause of cardiac arrest.

## 1. Introduction

Pulmonary embolism (PE) is regarded as an elusive diagnosis with a non-specific clinical presentation and has a tendency to be both over- and underdiagnosed in clinical practice [1, 2]. In the United States of America, venous thromboembolism (VTE) has been reported as the 3rd commonest cause of mortality [3]. Most patients with PE are clinically asymptomatic. In fact PE has been shown to be present in 60–80% of individuals with confirmed deep vein thrombosis (DVT), despite absence of symptom in more than half of these patients [4]. Cardiac arrest following PE has an associated mortality of up to 70% within the first hour of presentation [5] and an overall mortality of up to 95% [6]. Approximately 90% of episodes of cardiac arrests occur within 1-2 hours after the onset of symptoms of PE [7, 8].

Barring a handful of case reports and a few small and predominantly retrospective studies, no randomized controlled trial has thus far focused on the management of presumed PE in cardiac arrest victims. In this manuscript we present a case of neurologically intact survival after successful thrombolysis during cardiac arrest secondary to PE. We also discuss current guidelines and recommendations.

## 2. Case Report

**Figure 1:**
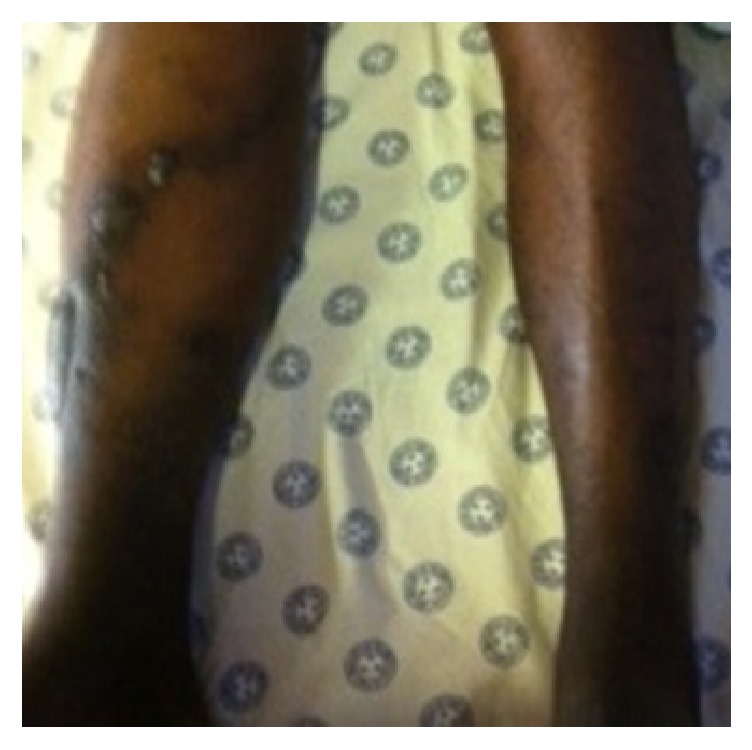
The right lower limb was noted to be larger than the left lower limb on arrival to the Emergency Department.

**Figure 2:**
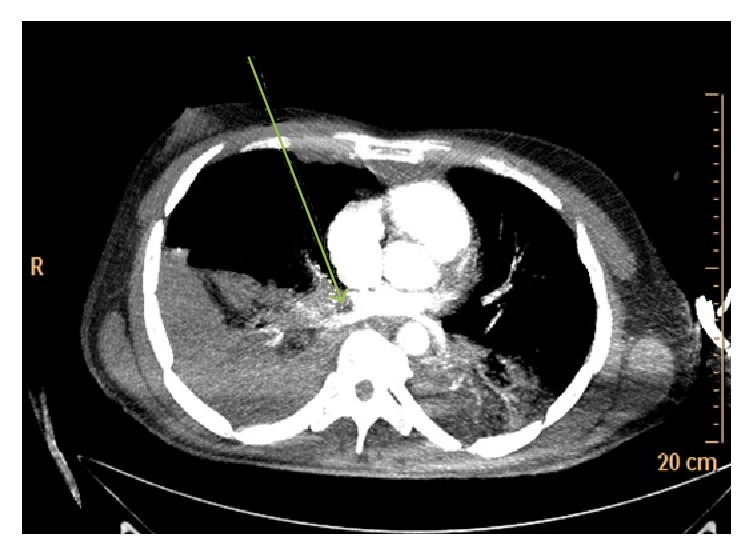
Coronal slice contrast enhanced computed tomography pulmonary angiography. The arrow demonstrates a large filling defect (pulmonary embolus) in the right pulmonary artery with an oligaemic right lung field.

**Figure 3:**
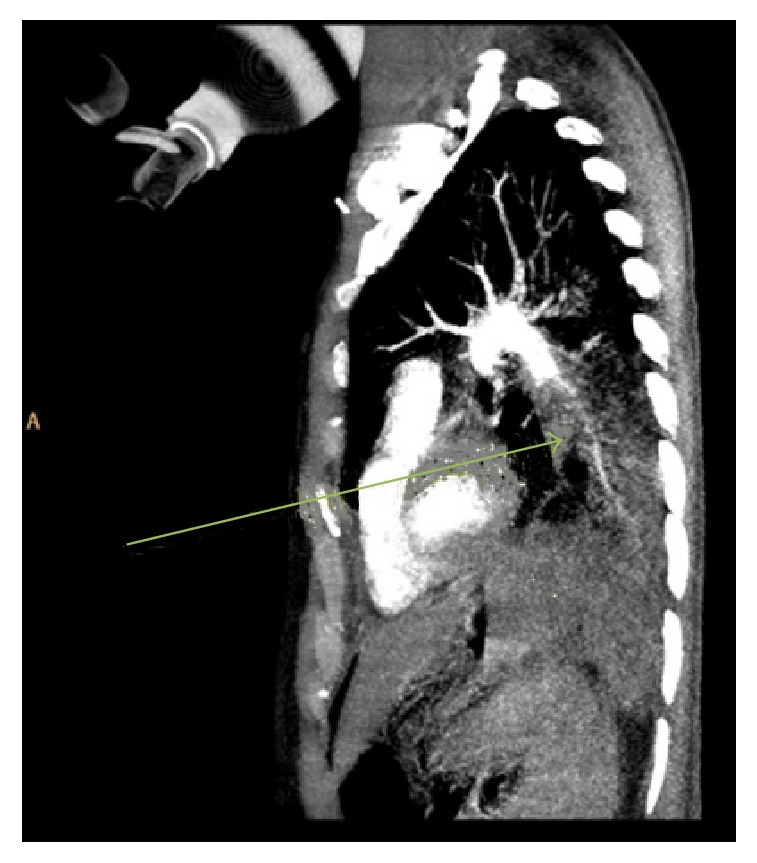
Left parasagittal contrast enhanced computed tomography pulmonary angiography. The arrow points to a large filling defect (pulmonary embolus) in the left pulmonary artery.

A 43-year-old man was rushed into the Emergency Department (ED) whilst undergoing cardiopulmonary resuscitation (CPR). Witnessed cardiac arrest had occurred during transportation to the hospital for sudden onset of shortness of breath. His initial cardiac rhythm on ED arrival was recorded as pulseless electrical activity (PEA). After confirming correct endotracheal tube placement, he was noted to have significant swelling of his entire right lower limb (Figure 1). A right sided iliofemoral DVT and dilatation of the right ventricle were demonstrated on point of care ultrasound assessment that had been performed in the ED. Thrombolytic therapy with alteplase (Actilase®, Boehringer Ingelheim Pharma GmbH & Co, Germany), 100 mg (10 mg over 1 minute and then 90 mg over 2 hours), was promptly initiated whilst high quality CPR was continued. A return of spontaneous circulation (ROSC) was achieved approximately 30 minutes later. The patient remained haemodynamically stable and was sent to the radiology department for a computed tomography pulmonary angiogram (CTPA) after completion of the alteplase infusion. The diagnosis of PE was confirmed on CTPA, which demonstrated multiple large filling defects in both main pulmonary arteries as well as regions of pulmonary oligaemia. An area of hyperdensity in the posterior right lung region, suggestive of an intra-alveolar haemorrhage, was also noted on CTPA as well as on the post-thrombolysis but not the pre-thrombolysis chest X-ray films (Figures 2 and 3). Since this did not result in any haemodynamic or ventilatory instability, it was decided to manage the intra-alveolar haemorrhage conservatively. Continuous monitoring of vital signs, including temperature, was maintained in the ICU to ensure that core body temperature remained below 37°C. There was no need to initiate any active cooling measures. On day 3 after admission, a repeat in cardiac echocardiography demonstrated that the right ventricle had normalized in size. Further diagnostic investigations led to the conclusion that enlargement of regional inguinal lymph nodes secondary to active venereal disease precipitated the development of the lower limb DVT. Seven days after thrombolysis and treatment of his underlying pathology, the patient had completely recovered and was discharged home neurologically intact.

## 3. Discussion

High quality chest compressions, early defibrillation, and correction of the underlying cause of cardiac arrest are the only three interventions that have been shown to improve outcomes in victims of cardiac arrest. Pulmonary thromboembolism, a well-known and reversible cause of cardiac arrest, is responsible for up to 9% of all cases of cardiac arrest [9, 10]. Other common reversible causes of cardiac arrest include hypoxaemia, potassium, and other electrolyte disturbances, hypo- or hyperthermia, acidosis, circulatory shock, tension pneumothorax, cardiac tamponade, myocardial infarction, and various toxins [11].

Pulmonary embolism as a cause of cardiac arrest is frequently missed. A post-mortem study by Kürkciyan and colleagues reported that PE as a cause of cardiac arrest was missed in 30% of all PE related cardiac arrest cases [12], whereas another autopsy study that included 16 postoperative patients documented the presence of PE in 62.5% of these patients [13].

External femoral vein obstruction and stasis was considered to be the cause of VTE in our patient. However, up to 30% of patients presenting with PE have no underlying risk factors [7], and many cardiac arrest victims have not complained of any preceding symptoms [14]. Although a unilateral swollen leg is highly suggestive of PE as a cause of cardiac arrest [15], approximately 30% of VTE's do not originate in the lower limbs. Furthermore, lower limb DVT's can also be found in individuals without any clinical features of DVT (silent DVT) [16].

Point of care ultrasonography has gained increasingly importance in the Emergency Department. It is a useful tool to screen for the presence of DVT and other indicators of PE [17, 18]. The presence of a thrombus in the lower limbs can be accurately determined by the trained ED clinician [19, 20]. In the cardiac arrest victim, although lacking sensitivity and specificity, echocardiographic evidence of an enlarged right ventricle with a flattened interventricular septum during routine pulse check intervals supports the diagnosis of PE [21, 22]. When available, a low end tidal CO2 reading, measured on quantitative waveform capnography during good quality CPR, may also support the diagnosis of PE [23]. Once the patient has been stabilised after ROSC, the diagnosis of PE can be confirmed with CTPA before complete breakdown of the thrombus has been achieved.

With regard to the implementation of therapeutic hypothermia in the post-cardiac arrest victim, previous guidelines have suggested a targeted temperature of 32–34°C [24]; however Nielsen and colleagues demonstrated that there was no additional benefit in maintaining the body temperature at 36°C (lower end of normal range) versus 33°C [25].

Overall there is a lack of good data supporting the use of thrombolysis in CPR [26]. However, major societal bodies have recommended its use when PE is either known or suspected as the cause of cardiac arrest [11, 27]. In the TROICA study, which is the only randomized controlled trial that compared fibrinolytic therapy to placebo in patients with out-of-hospital cardiac arrest of all causes, 525 out of 1050 patients received tenecteplase. The study was prematurely terminated due to futility. There were no significant differences in 30-day survival, hospital admission, ROSC, 24 hr survival, survival to hospital discharge, or neurologic outcome between the fibrinolysis and placebo groups. However, more cases of intracranial haemorrhage had been noted in the intervention group. Pulmonary embolism as a primary cause of cardiac arrest was only confirmed in 37/1050 patients. Since this group was underpowered, a significant 30-day survival benefit of thrombolytic treatment could not be demonstrated [28]. Contrastingly, a meta-analysis, which included patients with PE as a cause of cardiac arrest, concluded that fibrinolytic therapy was associated with an increase in ROSC, survival to hospital discharge, and better long term neurologic outcomes [29].

Although we infused the dose of alteplase over 2 hours as per the manufacturer's recommendation [30], others have recommended a more rapid infusion strategy (e.g., a 50 mg bolus injection of alteplase with an option for a repeat bolus 15 minutes later or an accelerated regimen of 0,6 mg alteplase/kg over 15 minutes) [31, 32].

One of the drawbacks of thrombolytic therapy includes the risk of potential intracranial and other major bleeding episodes. In a study by Janata et al. that included 66 patients with cardiac arrest secondary to PE, major bleeding complications occurred in 25% of patients who received thrombolytic therapy. There was no difference in bleeding complications between patients who had undergone CPR of more than 10 minutes duration compared to those that required less than 10 minutes of CPR. Although not statistically significant, survival to hospital discharge was also higher in the fibrinolytic therapy group (19% versus 7%) [33]. Considering the overall poor prognosis associated with cardiac arrest following PE, the benefits of thrombolysis may still outweigh the risk of major bleeding.

Other therapeutic options include surgical embolectomy and percutaneous mechanical thrombectomy [11, 27]. The evidence for surgical thrombectomy and percutaneous mechanical thrombectomy during CPR is weak [11]. In a retrospective study that included 13 patients who had undergone surgical embolectomy for massive PE, only 1 out of the 4 patients (25%) with cardiac arrest survived. There were no in-hospital deaths amongst patients without cardiac arrest [34]. In another small study by Fava and colleagues, 86% of subjects survived after percutaneous mechanical thrombectomy was performed during CPR.

In conclusion, considering that outcomes after cardiac arrest are generally dismal and that fibrinolytic therapy is a potentially life-saving intervention, the ED clinician must not hesitate to administer thrombolytics whenever PE is considered the most likely cause of cardiac arrest.
